# Asthma susceptible genes in Chinese population: A meta-analysis

**DOI:** 10.1186/1465-9921-11-129

**Published:** 2010-09-24

**Authors:** Xiaobo Li, Yonggang Zhang, Jie Zhang, Yuling Xiao, Jin Huang, Can Tian, Chao He, Yao Deng, Yingying Yang, Hong Fan

**Affiliations:** 1 Department of Respiratory Medicine, The 452nd Military Hospital of China, Chengdu, Sichuan 610041, China; 2 Department of Respiratory Medicine, West China Hospital of Sichuan University, Chengdu, Sichuan 610041, China; 3 Zhejiang Provincial Key Laboratory of Medical Genetics, Wenzhou Medical College, Wenzhou, Zhejiang, 325035, China; 4 Department of Laboratory Medicine, West China Hospital of Sichuan University, Chengdu, Sichuan 610041, China; 5 West China Medical School/West China Hospital, Sichuan University, Chengdu, Sichuan 610041, China; 6 Chinese Evidence-Based Medicine/Cochrane Center, Chengdu, Sichuan 610041, China

## Abstract

**Background:**

Published data regarding the associations between genetic variants and asthma risk in Chinese population were inconclusive. The aim of this study was to investigate asthma susceptible genes in Chinese population.

**Methods:**

The authors conducted 18 meta-analyzes for 18 polymorphisms in 13 genes from eighty-two publications.

**Results:**

Seven polymorphisms were found being associated with risk of asthma, namely: *A Disintegrin and Metalloprotease 33 *(*ADAM33*) T1-C/T (odds ratio [OR] = 6.07, 95% confidence interval [CI]: 2.69-13.73), *Angiotensin-Converting Enzyme *(*ACE*) D/I (OR = 3.85, 95%CI: 2.49-5.94), *High-affinity IgE receptor β chain *(*FcεRIβ*) -6843G/A (OR = 1.49, 95%CI: 1.01-2.22), *Interleukin 13*(*IL-13*) -1923C/T (OR = 2.99, 95%CI: 2.12-4.24), *IL-13 *-2044A/G (OR = 1.49, 95%CI: 1.07-2.08), *Regulated upon Activation, Normal T cell Expressed and Secreted *(*RANTES*) -28C/G (OR = 1.64, 95%CI: 1.09-2.46), *Tumor Necrosis Factor-α *(*TNF-α*) -308G/A(OR = 1.42, 95%CI: 1.09, 1.85). After subgroup analysis by age, the *ACE *D/I, *β2-Adrenergic Receptor *(*β2-AR*) -79G/C, *TNF-α *-308G/A, *Interleukin 4 receptor*(*IL-4R*) -1902G/A and *IL-13 *-1923C/T polymorphisms were found significantly associated with asthma risk in Chinese children. In addition, the *ACE *D/I, *FcεRIβ *-6843G/A, *TNF-α *-308G/A, *IL-13 *-1923C/T and *IL-13 *-2044A/G polymorphisms were associated with asthma risk in Chinese adults.

**Conclusion:**

*ADAM33, FcεRIβ, RANTES, TNF-α, ACE, β2-AR, IL-4R *and *IL-13 *genes could be proposed as asthma susceptible genes in Chinese population. Given the limited number of studies, more data are required to validate these associations.

## Introduction

Asthma is one of the most common chronic respiratory diseases, affecting about 300 millions of children and adults worldwide[1]. In China, more than 25 millions people are asthmatic patients, which includes almost 10 million children[2]. Compared with the western world, the preventive controls and treatments for asthma were not well established in China [3]. Only a few percent of asthma patients received proper treatment. Poverty and inadequate resources are the main hindrance to reduce the burden of disease in China especially in numerous of Chinese villagers. Therefore, the best approach to reduce asthma is primary prevention through modifying the risk factors of asthma.

It is well accepted that asthma is a complex disease and both genetic and environmental factors contribute to its inception and evolution[4,5]. Many studies regarding associations between genetic variants and asthma risk have been published and many genes were proposed as asthma susceptible genes[6-9]. However, the conclusions obtained from different populations were often different or even controversial. Possible roles may be that different genetic backgrounds and environment exposures in different ethnic population that may affect the pathogenesis of asthma. Thus, asthma susceptible genes in different population may not be the same.

In recent years, host genetic susceptibility to asthma has been a research focus in scientific community in China. Many genes were suggested as asthma risk factors for Chinese population; however, many of the studies drew incompatible or even contradictory results. Considering a small number of sample size may be lack of power to reveal the reliable conclusion, we carried out a meta-analysis to assess the susceptible genes for asthma in Chinese population. To our knowledge, this is the first comprehensive and largest genetic meta-analysis conducted in people of Chinese descent for any respiratory diseases.

## Materials and methods

### Literature search

We conducted a literature search by using the electronic database Medline (Ovid), Pubmed, Embase, ScienceDirect, Springer, CNKI, Wanfang database, Weipu database and CBM database to identify articles that evaluated the association between genetic variants and the risk of asthma in Chinese population (Last search was updated on May 13, 2010). The search terms were used as follows: '*asthma *or *asthmatic*', in combination with '*polymorphism *or *variant *or *mutation*' and '*Chinese *or *China*' for Medline (Ovid), Pubmed, Embase, ScienceDirect, Springer database; '*asthma *or *asthmatic*', in combination with '*polymorphism *or *variant *or *mutation*' for CNKI database, Wanfang database, Weipu database and CBM database. All languages were included. The following criteria were used for selecting literatures in the meta-analysis: (1) the study should evaluate the association between genetic variants and risk of asthma in Chinese population from either mainland, overseas or both, (2) the study should be a case-control design published in a journal (3) genotype distributions in both cases and controls were available for estimating an odds ratio with 95% confidence interval (CI) and *P *value, (4)genotype distributions of control population must be consistent with Hardy-Weinberg equilibrium(HWE), *P *> 0.05 (5) the polymorphism for data synthesis should be studied in at least three case-control studies, (6) polymorphisms for data synthesis should be characterized as -A/B, with the following genotypes: AA, AB and BB. Accordingly, the following exclusion criteria were used: (1) abstracts and reviews, (2)genotype frequency not reported, (3) repeated or overlapping publications (4) polymorphisms with data less than three case-control studies (5) genotype distributions of control population not consistent with HWE, (6)genetic variants not characterized as -A/B. For duplication or overlapping publications, the studies with larger number of cases and controls or been published latest were included.

### Data extraction

Two independent authors (Xiaobo Li and Yonggang Zhang) checked all potentially relevant studies and reached a consensus on all items. In case of disagreement, a third author(Jie Zhang) would assess these articles. The following data were collected from each study: first author, year of publication, location of the people, ages, genotype frequencies in cases and controls.

### Statistical Analysis

For each case-control study, we first examined whether the genotype distribution in control group was according to Hardy-Weinberg equilibrium by Pearson's *X^2 ^*test http://ihg2.helmholtz-muenchen.de/cgi-bin/hw/hwa1.pl.

Any polymorphism that had been studied in at least three case-control studies was included in the meta-analysis. The strength of the associations between asthma risk and genetic variants were estimated by ORs and 95% CIs. The statistical significance of summary ORs were assessed by Z-test. The evaluated genetic models for each study were based mostly on those used in primary studies. Heterogeneity was evaluated by a *X^2^*-based *Q *statistic and was considered statistical significant at *P *value < 0.10. *I^2 ^*was used to measure the percentage of variability in point estimated that due to heterogeneity rather than sampling error. When the *P*-value is > 0.10, the pooled OR was calculated by the fixed-effects model, otherwise, a random-effects model was used. To evaluate the age-specific effects, subgroup analyses were performed by age for polymorphisms which were investigated in a sufficient number of studies(data were available from at least three case-control studies for at least one subgroup). Publication bias was examined by using the funnel plots, Begg's test and Egger's test[4]. The funnel plot is asymmetrical when there is evidence of publication bias. All statistical tests were performed by using REVMAN 4.2 software and STATA 10.0.

## Results

### Candidate asthma-genes in Chinese Population

**Figure 1:**
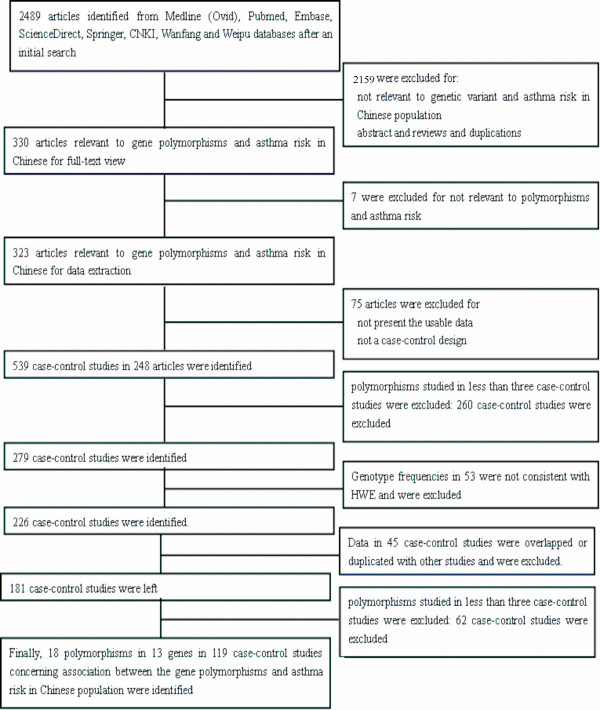
**Flow diagram of included/excluded studies**.

The selection process is shown in Figure 1. Briefly, 2489 search results were identified from Medline (Ovid), Pubmed, Embase, ScienceDirect, Springer, CNKI database, Wanfang database, Weipu database and CBM database in the initial search. After reading the titles and abstracts, 2159 articles were excluded for abstracts, reviews, duplicated search results or not being relevant to genetic variants and asthma risk in Chinese population. By reading through the full texts of the remaining 330 articles, 7 articles were excluded for not being relevant to polymorphisms and asthma risk. The remaining 323 articles were used for data extraction. A total of 539 case-control studies were extracted from 248 articles, and 75 articles were excluded because of the absence of the usable data or not a case-control design. In meta-analysis, a small number of studies weaken the conclusions; therefore, only polymorphisms which had been investigated in at least three case-control studies were included for data synthesis. Thus, we excluded all these polymorphisms which were studied in less than three case-control studies(a total of 260 case-control studies were excluded). Hence, a total of 279 case-control studies were left. In addition, genotype frequencies for control population in 53 case-control studies were not consistent with HWE and these case-control studies were all excluded. In the remaining 226 case-control studies, data in 45 case-control studies were overlapped or duplicated with other studies and these case-control studies were all excluded. Thus, 181 case-control studies were left. Among the 181 case-control studies, some polymorphisms were studied in less than three case-control studies, and these polymorphisms were also excluded(a total of 62 case-control studies were excluded). Finally, a total of 18 polymorphisms in 13 genes in 119 case-control studies concerning genetic variants and asthma risk in Chinese population met the inclusion criteria, were identified for data synthesis (Table 1). The characteristics of each polymorphism are listed in Table 2, 3, 4, 5, 6, 7, 8, 9, 10, 11, 12, 13, 14, 15, 16, 17, 18 and 19. The genetic models for pooling data are also listed in Table 1.

**Table 1:** Genes identified from individual studies

Gene	Chromosome location of gene	Polymorphism	Aminoacid change	Genetic model	Genotypes Evaluated	Other genotypes	Cases	Controls
β2-AR	5q31-32	-46G/A	Arg16Gly	Recessive	GG	GA+AA	1796	1589
		-79G/C	Gln27Glu	Recessive	GG	GC+CC	823	692
IL-4R	16p11.2-12.1	-1902G/A	Q576R	Dominant	GG+GA	AA	2308	1971
		-223G/A	Ile/Val	Recessive	GG	GA+AA	1623	1304
IL-4	5q31	-589C/T		Dominant	CC+CT	TT	1724	1656
TNF-α	6p21.1-21.3	-308A/G		Dominant	AA+AG	GG	1428	1511
FcεRIβ	11q13	-6843G/A	Glu237Gly	Dominant	GG+GA	AA	1434	1276
		-109C/T		Recessive	CC	CT+TT	428	371
ACE	17q23	D/I		Recessive	DD	DI+II	385	335
IL-13	5q31	-2044A/G	Gln130Arg	Dominant	AA+AG	GG	1512	1351
		-1923C/T		Recessive	TT	CC+CT	645	588
IL-1β	2q12-21	-511C/T		Dominant	TT+TC	CC	333	255
LT-α	6q21.3	+252A/G		Dominant	GG+GA	AA	674	896
TGF-β1	19q13	-509C/T		Dominant	TT+TC	CC	406	390
CD14	5q31.1	-159C/T		Dominant	TT+TC	CC	1381	1219
ADAM33	20p13	T1	Met764Thr	Recessive	CC	TT+TC	569	512
RANTES	17q11.2-12	-28G/C		Dominant	GG+GC	CC	314	229
		-403A/G		Dominant	AA+AG	GG	343	205

**Table 2:** Main data of all studies included in the meta-analysis for the -46G/A (Arg16Gly) polymorphism in β2-AR gene

				Case			Control				
						
Study	Population location	Year	Age	AA	AG	GG	AA	AG	GG	OR	95%CI
Chan, I H[16]	Hong Kong	2008	10.4 ± 3.7	101	135	59	51	89	33	1.06	0.66-1.70
Cui, LY(Han)[17]	Neimenggu	2007	21-62	6	34	2	6	20	4	0.33	0.06-1.90
Cui, LY(Meng)[17]	Neimenggu	2007	26-69	3	21	6	6	19	5	1.25	0.34-4.64
Gao, J M[18]	Beijing	2004	38.7 ± 13.8	38	59	28	35	53	8	3.18	1.37-7.33
Li, H[19]	Shanghai	2009	3-12	86	76	30	46	100	46	0.59	0.35-0.98
Liao, W[20]	Chongqing	2001	5.8 ± 4.3	12	27	11	14	28	8	1.48	0.54-4.06
Qiu, Y Y(2008)[21]	Jiangsu	2008	63.2 ± 5.6	25	31	14	34	55	23	0.97	0.46-2.04
Shi, X H[22]	Jiangsu	2008	34(14-66)	22	19	7	10	25	13	0.46	0.17-1.28
Wang, Z[23]	Anhui	2001	30.6 ± 16.2	52	54	22	38	64	34	0.62	0.34-1.14
Xie, Y[24]	Shanghai	2008	4.98 ± 2.78	14	37	6	21	34	7	0.92	0.29-2.93
Xing, J[25]	Beijing	2001	20-66	9	62	29	29	55	16	2.14	1.08-4.26
Zhang, X Y[26]	Chongqing	2008	1.08-17	81	111	25	19	23	8	0.68	0.29-1.62
Wang, J Y[27]	Taiwan	2009	7.82 ± 3.81	138	207	97	173	250	87	1.37	0.99-1.89

**Table 3:** Main data of all studies included in the meta-analysis for the -79G/C (Gln27Glu) polymorphism in *β2-AR *gene

				Case			Control				
						
Study	Population location	Year	Age	CC	CG	GG	CC	CG	GG	OR	95%CI
Cui, LY(Han)[17]	Neimenggu	2007	21-62	32	6	4	26	3	1	3.05	0.32-28.79
Gao, G K[28]	Beijing	2002	4-56	20	32	6	32	49	8	1.17	0.38-3.56
Liao, W[20]	Chongqing	2001	5.8 ± 4.3	26	20	4	20	27	3	1.36	0.29-6.43
Lin, Y C[29]	Taiwan	2003	13.9 ± 0.07	65	15	0	54	14	1	0.28	0.01-7.08
Pan, Y P[30]	Jiangxi	2005	-	15	24	6	17	23	5	1.23	0.35-4.37
Qiu, Y Y(2000)[31]	Jiangsu	2000	42 ± 5	23	30	6	29	36	7	1.05	0.33-3.32
Qiu, Y Y(2008)[21]	Jiangsu	2008	63.2 ± 5.6	56	13	1	90	20	2	0.80	0.07-8.96
Wang, Z[23]	Anhui	2001	30.6 ± 16.2	108	19	1	113	22	1	1.06	0.07-17.18
Ye, X W[32]	Guizhou	2003	42.68 ± 10.55	25	39	10	15	20	4	1.37	0.40-4.68
Zhang, X Y[26]	Chongqing	2008	1.08-17	54	119	44	8	24	18	0.45	0.23-0.88

**Table 4:** Main data of all studies included in the meta-analysis for the -1902G/A (Q576R) polymorphism in *IL-4R *gene

				Case			Control				
						
Study	Population location	Year	Age	AA	AG	GG	AA	AG	GG	OR	95%CI
Cui, T P[33]	Hubei	2003	3-68	129	89	23	130	41	4	2.51	1.64-3.83
Deng, R Q[34]	Guangdong	2006	8-75	26	42	32	15	38	47	0.50	0.25-1.02
Gui, Q[35]	Chongqing	2006	49(28-72)	33	15	2	34	14	2	1.09	0.48-2.52
Hu, S Y[36]	Guangdong	2005	2-16	90	66	19	130	41	4	2.73	1.74-4.28
Liu, L N[37]	Henan	2005	3-15	46	27	3	47	12	1	2.36	1.09-5.08
Mak, J C[38]	Hong Kong	2007	42.4 ± 16.1	200	81	4	191	91	9	0.81	0.57-1.15
Sun, J[39]	Heilongjiang	2010	3-14	67	24	0	33	9	0	1.31	0.55-3.14
Wu, X H[40]	Hubei	2010	8.8	183	61	8	168	55	4	1.07	0.72-1.61
Zhang, A M[41]	Hunan	2005	3-14	55	39	0	57	11	0	3.67	1.71-7.89
Zhang, H[42]	Shanghai	2007	-	257	87	8	87	27	0	1.19	0.73-1.95
Zhang, W[43]	Singapore	2007	-	115	30	0	115	38	4	0.71	0.42-1.22
Wang, J Y[27]	Taiwan	2009	7.82 ± 3.81	326	112	9	360	140	12	0.88	0.66-1.17

**Table 5:** Main data of all studies included in the meta-analysis for the -223G/A (Ile/Val) polymorphism in *IL-4R *gene

				Case			Control				
						
Study	Population location	Year	Age	AA	AG	GG	AA	AG	GG	OR	95%CI
Chan, I H [16]	Hong Kong	2008	10.4 ± 3.7	79	159	57	49	80	38	0.81	0.51-1.29
Deng, R Q[44]	Guangdong	2006	8-75	24	47	29	9	33	58	0.30	0.16-0.53
Yang, Q[45]	Jiangxi	2004	18-71	6	21	7	8	16	5	1.24	0.35-4.44
Zhang, H[42]	Shanghai	2007	-	106	168	78	44	53	17	1.62	0.92-2.88
Zhang, W[43]	Singapore	2007	-	32	84	29	42	76	39	0.76	0.44-1.30
Wang, J Y[27]	Taiwan	2009	7.82 ± 3.81	105	201	139	124	250	136	1.25	0.94-1.65
Wu, X H[40]	Hubei	2010	8.8	46	131	75	59	110	58	1.23	0.83-1.85

**Table 6:** Main data of all studies included in the meta-analysis for the -589 C/T polymorphism in *IL-4 *gene

				Case			Control				
						
Study	Population location	Year	Age	TT	CT	CC	TT	CT	CC	OR	95%CI
Cui, T P[33]	Hubei	2003	3-68	141	89	11	114	52	9	1.33	0.89-1.98
Hu, S Y[36]	Guangdong	2005	2-16	108	59	8	114	52	9	1.16	0.75-1.79
Liu, L N[37]	Henan	2005	3-15	45	29	2	34	23	3	0.90	0.45-1.79
Mak, J C[38]	Hong Kong	2007	42.4 ± 16.1	179	95	15	186	87	19	1.08	0.77-1.51
Wang, W[46]	Xinjiang	2004	39 ± 8	22	42	29	15	26	21	1.03	0.49-2.19
Wu, X H[40]	Hubei	2010	8.8	163	83	6	132	84	11	0.76	0.52-1.10
Zhang, W D[47]	Singapore	2005	-	101	47	4	109	45	3	1.15	0.71-1.85
Wang, J Y[27]	Taiwan	2009	7.82 ± 3.81	279	145	22	309	183	16	0.93	0.72-1.21

**Table 7:** Main data of all studies included in the meta-analysis for the -308A/G polymorphism in TNF-α gene

				Case			Control				
						
Study	Population location	Year	Age	GG	GA	AA	GG	GA	AA	OR	95%CI
Gao, J M[48]	Beijing	2003	38.7 ± 13.8	47	52	26	44	41	11	1.40	0.82-2.41
Guo, Y L[49]	Jiangxi	2004	-	4	28	16	7	11	3	5.50	1.40-21.60
Li, Z F[50]	Guangdong	2003	2-12	9	16	5	14	10	2	2.72	0.91-8.16
Liu, R M[51]	Hubei	2004	2-15	98	15	0	104	22	0	0.72	0.36-1.47
Mak, J C[38]	Hong Kong	2007	42.4 ± 16.1	244	47	1	250	40	2	1.17	0.75-1.84
Tan, E C[52]	Singapore	1999	-	49	18	0	115	36	0	1.17	0.61-2.26
Wang, T N[53]	Taiwan	2004	5-18	140	49	2	111	18	0	2.25	1.24-4.06
Zhai, F Z[54]	Shandong	2004	35.80 ± 10.18	44	14	6	67	12	1	2.34	1.06-5.19
Zhao, H J[55]	Jilin	2005	-	45	5	0	71	9	0	0.88	0.28-2.78
Wang, J Y[27]	Taiwan	2009	7.82 ± 3.81	345	100	3	409	94	7	1.21	0.89-1.65

**Table 8:** Main data of all studies included in the meta-analysis for the -6843G/A polymorphism in *FcεRI β *gene

				Case			Control				
						
Study	Population location	Year	Age	AA	AG	GG	AA	AG	GG	OR	95%CI
Chan, I H[16]	Hong Kong	2008	10.4 ± 3.7	267	23	1	154	13	0	1.06	0.53-2.15
Cui, T P[56]	Hubei	2004	40.37 ± 15.09	60	40	6	78	26	2	2.14	1.20-3.81
Liu, T[57]	Shandong	2006	36.5	45	14	1	39	10	1	1.18	0.49-2.87
Tang, Y[58]	Guangdong	2003	39.5(12-67)	49	11	0	61	4	0	3.42	1.03-11.42
Wang, L[59]	Hubei	2003	2-16	65	40	5	70	20	2	2.20	1.20-4.06
Zeng, L X[60]	Jiangxi	2001	37(14-63)	61	5	3	27	1	0	3.54	0.42-29.73
Zhang, X Z[61]	Singapore	2004	52 ± 16	81	57	3	108	42	7	1.63	1.02-2.62
Zhao, K S[62]	Jilin	2004	1.5-14	126	23	2	92	13	0	1.40	0.68-2.89
Wang, J Y[27]	Taiwan	2009	7.82 ± 3.81	309	121	16	314	165	27	0.73	0.55-0.95

**Table 9:** Main data of all studies included in the meta-analysis for the -109C/T polymorphism in FcεRI β gene

				Case			Control				
						
Study	Population location	Year	Age	TT	TC	CC	TT	CT	CC	OR	95%CI
Li, H[19]	Shanghai	2009	3-12	110	58	24	78	90	24	1.00	0.55-1.83
Wang, L[59]	Hubei	2003	2-16	43	54	13	35	46	11	0.99	0.42-2.32
Zhao, K S [63]	Jilin	2004	5.6 ± 3.1	46	69	11	40	38	9	0.83	0.33-2.09

**Table 10:** Main data of all studies included in the meta-analysis for the D/I polymorphism in ACE gene

				Case			Control				
						
Study	Population location	Year	Age(year)	II	DI	DD	II	DI	DD	OR	95%CI
Gao, J M[64]	Beijing	1999	39(16-69)	12	15	23	16	26	8	4.47	1.75-11.43
Guo, Y B[65]	Guangdong	2006	0.33-3	27	18	7	36	32	4	2.64	0.73-9.56
Lu, H M[66]	Tianjin	2004	37(18-52)	3	4	11	5	7	3	6.29	1.29-30.54
Lue, K H[67]	Taiwan	2006	9.91 ± 1.62	48	40	17	56	42	4	4.73	1.53-14.60
Qin, J H[68]	Liaoning	2000	6.9 ± 2.7	24	10	18	21	14	5	3.71	1.24-11.10
Song, L J[69]	Jilin	2001	1-14	22	45	41	18	29	9	3.20	1.42-7.20

**Table 11:** Main data of all studies included in the meta-analysis for the -2044A/G polymorphism in IL-13 gene

				Case			Control				
						
Study	Population location	Year	Age	GG	AG	AA	GG	AG	AA	OR	95%CI
Chan, I H[16]	Hong Kong	2008	10.4 ± 3.7	94	136	43	54	70	17	1.18	0.78-1.80
Feng, D[70]	Heilongjiang	2009	3-16	17	18	10	30	10	3	3.80	1.57-9.23
Liu, J L[71]	Guangdong	2004	14-67	27	54	19	44	46	10	2.12	1.17-3.84
Wu, X H[40]	Hubei	2010	8.8	105	111	36	125	84	18	1.72	1.19-2.46
Yang, L F[72]	Gansu	2010	8 ± 4	71	60	47	73	66	19	1.29	0.84-2.00
Zhao, K S[73]	Jilin	2005	1.5-14	18	60	52	8	42	50	0.54	0.23-1.30
Wang, J Y[27]	Taiwan	2009	7.82 ± 3.81	203	194	49	212	234	59	0.87	0.67-1.12
Xi, D[74]	Hubei	2004	≥20	15	24	6	23	20	3	2.08	1.28-3.38
Xi, D[74]	Hubei	2004	≥4	10	25	8	16	13	2	3.52	1.30-9.55

**Table 12:** Main data of all studies included in the meta-analysis for the -1923C/T polymorphism in IL-13 gene

				Case			Control				
						
Study	Population location	Year	Age	CC	CT	TT	CC	CT	TT	OR	95%CI
Song, Q Z[75]	Guangdong	2005	14-67	24	55	21	43	47	10	2.39	1.06-5.39
Shi, X H[22]	Jiangsu	2008	34(14-66)	12	26	10	30	16	2	6.05	1.25-29.32
Chen, J Q[76]	Jiangsu	2004	2.59 ± 1.45	41	43	12	39	14	0	15.83	0.92-272.92
Wang, X H[77]	Shandong	2009	39 ± 11	31	57	61	66	68	26	3.57	2.10-6.08
Wu, X H[40]	Hubei	2010	8.8	106	114	32	126	85	16	1.92	1.02-3.60

**Table 13:** Main data of all studies included in the meta-analysis for the -511C/T polymorphism in IL-1β gene

				Case			Control				
						
Study	Population location	Year	Age	GG	GA	AA	GG	GA	AA	OR	95%CI
Hsieh, C C[78]	Taiwan	2004	8.74 ± 4.09	69	93	40	48	70	26	0.96	0.61-1.52
Wu, Z F[79]	Jiangxi	2007	11-68	16	36	24	26	38	12	1.95	0.94-4.03
Zhao, X F[80]	Yunnan	2006	5.9(3-14)	51	4	0	30	5	0	0.47	0.12-1.89

**Table 14:** Main data of all studies included in the meta-analysis for the +252A/G polymorphism in LT-α gene

				Case			Control				
						
Study	Population location	Year	Age	AA	AG	GG	AA	AG	GG	OR	95%CI
Gao, J M[81]	Beijing	2003	38.7 ± 13.8	13	63	49	14	46	36	1.47	0.66-3.30
Ma, W C[82]	Guangdong	2005	1.8-9	8	14	10	26	46	28	1.05	0.42-2.64
Mak, J C[38]	Hong Kong	2007	42.4 ± 16.1	70	146	69	79	134	76	1.16	0.80-1.68
Tan, E C[52]	Singapore	1999	-	13	38	15	30	84	39	0.99	0.48-2.06
Xu, X[83]	Guangdong	2003	18-69	12	21	19	26	47	30	1.13	0.51-2.46
Huang, S C[84]	Taiwan	2008	9.9 ± 4.1	20	69	25	45	69	41	1.62	0.98-2.66

**Table 15:** Main data of all studies included in the meta-analysis for the -509C/T polymorphism in *TGF-β1 *gene

				Case			Control				
						
Study	Population location	Year	Age	CC	CT	TT	CC	CT	TT	OR	95%CI
Lu, J R[85]	Jilin	2004	1-13	45	38	15	30	19	3	1.61	0.81-3.17
Mak, J C[86]	Hong Kong	2006	41.0 ± 16.1	46	109	93	51	155	102	0.87	0.56-1.35
Xia, W[87]	Jiangxi	2006	15-60	22	26	12	17	11	2	2.26	0.92-5.52

**Table 16:** Main data of all studies included in the meta-analysis for the -159C/T polymorphism in CD14 gene

				Case			Control				
						
Study	Population location	Year	Age	CC	CT	TT	CC	CT	TT	OR	95%CI
Chan, I H[16]	Hong Kong	2008	10.4 ± 3.7	55	134	80	26	77	38	0.88	0.52-1.48
Chen, M[88]	Guangdong	2009	14-71	63	62	25	40	68	42	0.50	0.31-0.82
Cui, T P[89]	Hubei	2003	2-16	27	67	49	10	42	20	0.69	0.32-1.52
Tan, C Y[90]	Taiwan	2006	-	17	56	47	24	55	41	1.51	0.77-2.99
Wu, X H[40]	Hubei	2010	8.8	54	117	81	31	121	75	0.58	0.36-0.94
Wang, J Y[27]	Taiwan	2009	7.82 ± 3.81	160	230	57	177	236	96	0.96	0.73-1.25

**Table 17:** Main data of all studies included in the meta-analysis for the T1-C/T polymorphism in *ADAM33 *gene

				Case			Control				
						
Study	Population location	Year	Age	TT	TC	CC	TT	TC	CC	OR	95%CI
Su, D J[91]	Heilongjiang	2008	36.69 ± 11.53	63	78	40	117	29	5	8.28	3.18-21.59
Wang, P[92]	Shandong	2006	43.32	250	45	1	236	33	1	0.91	0.06-14.65
Xiong, J Y[93]	Guangdong	2009	6-13	71	19	2	80	10	1	2.00	0.18-22.45

**Table 18:** Main data of all studies included in the meta-analysis for the -28G/C polymorphism in RANTES gene

				Case			Control				
						
Study	Population location	Year	Age	CC	CG	GG	CC	CG	GG	OR	95%CI
Liu, M[94]	Yunnan	2005	7.2 ± 4.8	25	6	1	29	3	0	2.71	0.63-11.59
Wang, L J[95]	Hubei	2004	9 ± 3	65	31	4	72	17	1	2.15	1.11-4.17
Yao, T C[96]	Taiwan	2003	-	134	39	9	83	23	1	1.24	0.71-2.17

**Table 19:** Main data of all studies included in the meta-analysis for the -403A/G polymorphism in RANTES gene

				Case			Control				
						
Study	Population location	Year	Age	GG	GA	AA	GG	GA	AA	OR	95%CI
Leung, T F[97]	Hongkong	2005	9.9 ± 3.4	60	53	16	37	21	8	1.47	0.81-2.66
Liu, M[94]	Yunnan	2005	7.2 ± 4.8	17	13	2	16	14	2	0.88	0.33-2.35
Yao, T C[96]	Taiwan	2003	-	98	65	19	60	41	6	1.09	0.68-1.77

### Summary results of Meta-analyzes

For each polymorphism, heterogeneity was analyzed by a *X^2^*-based *Q *statistic and was considered statistical significant at *P*-value <0.10. When the *P*-value is less than 0.10, the pooled OR of each meta-analysis was calculated by the fixed-effects model; otherwise, a random-effects model was used. The chosen models to synthesize the data for each polymorphism can be seen in Table 20.

**Table 20:** Summary results of the meta-analysis and publications bias

						Pubilication bias (Begg's test)	
	
Gene	Polymorphism	Genotype investigated	Studies Number	Effect Model	OR(95%CI)	t	*P*
β2-AR	-46G/A	GG	13	Random	1.02(0.75, 1.38)	-0.66	0.525
	-79G/C	GG	10	Fixed	0.86(0.58, 1.29)	1.60	0.148
IL-4R	-1902G/A	GG+GA	12	Random	1.30(0.94, 1.80)	0.92	0.377
	-223G/A	GG	7	Random	0.92(0.63, 1.35)	-0.81	0.453
IL-4	-589C/T	CC+CT	8	Fixed	1.01(0.88, 1.16)	0.53	0.615
TNF-α	-308A/G	AA+AG	10	Random	1.42(1.09, 1.85)	1.38	0.205
FcεRIβ	-6843G/A	GG+GA	9	Random	1.49(1.01, 2.22)	2.82	0.026
	-109C/T	CC	3	Fixed	0.96(0.62, 1.48)	-1.10	0.471
ACE	D/I	DD	6	Fixed	3.85(2.49, 5.94)	0.88	0.429
IL-13	-2044A/G	AA+AG	9	Random	1.49(1.07, 2.08)	1.93	0.095
	-1923C/T	TT	5	Fixed	2.99(2.12, 4.24)	1.19	0.320
IL-1β	-511C/T	TT+TC	3	Fixed	1.10(0.76, 1.59_	-0.16	0.896
LT-α	+252A/G	GG+GA	6	Fixed	1.26(0.98, 1.62)	-0.02	0.985
TGF-β1	-509C/T	TT+TC	3	Fixed	1.17(0.83, 1.64)	8.57	0.074
CD14	-159C/T	TT+TC	6	Random	0.79(0.59, 1.06)	-0.41	0.700
ADAM33	T1-C/T	CC	3	Fixed	6.07(2.69, 13.73)	-8.22	0.077
RANTES	-28G/C	GG+GC	3	Fixed	1.64(1.09, 2.46)	0.87	0.544
	-403A/G	AA+AG	3	Fixed	1.18(0.83, 1.67)	-0.37	0.777

**Figure 2:**
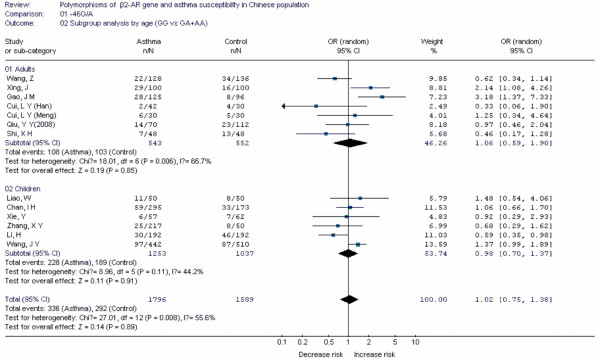
**Forest plot of asthma risk associated with *β2-AR *-46G/A in Chinese population**. Subgroup analysis by age.

**Figure 3:**
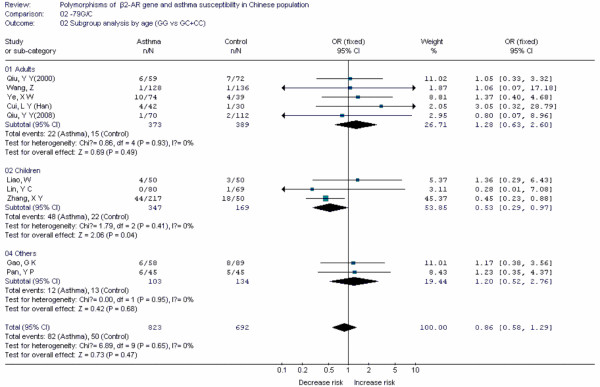
**Forest plot of asthma risk associated with *β2-AR *-79G/C in Chinese population**. Subgroup analysis by age.

**Figure 4:**
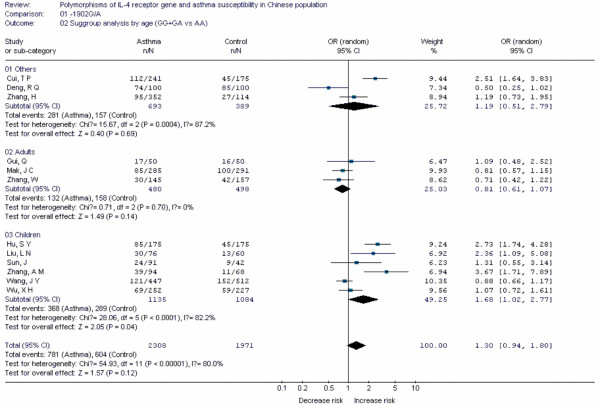
**Forest plot of asthma risk associated with *IL-4R *-1902G/A in Chinese population**. Subgroup analysis by age.

**Figure 5:**
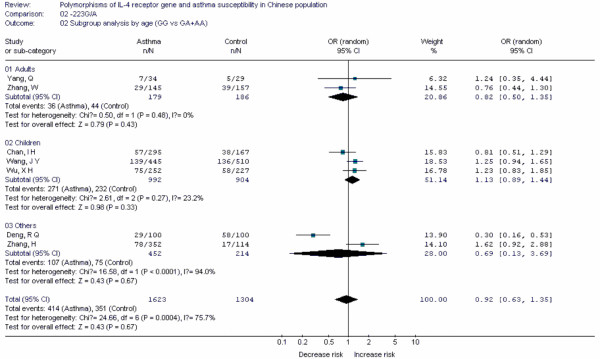
**Forest plot of asthma risk associated with *IL-4R *-223G/A in Chinese population**.

**Figure 6:**
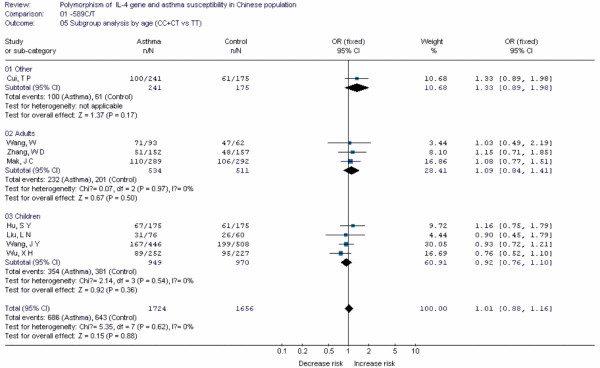
**Forest plot of asthma risk associated with *IL-4 *-589C/T in Chinese population**. Subgroup analysis by age.

**Figure 7:**
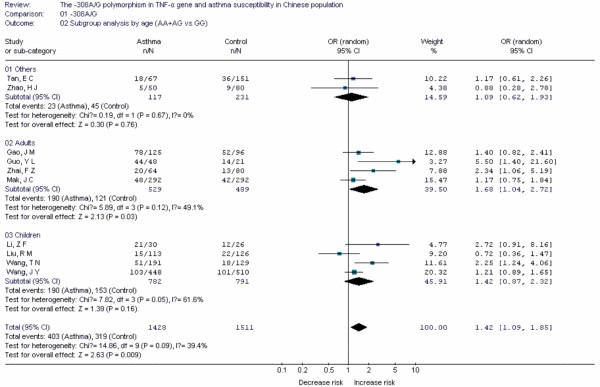
**Forest plot of asthma risk associated with *TNF-α *-308A/G in Chinese population**. Subgroup analysis by age.

**Figure 8:**
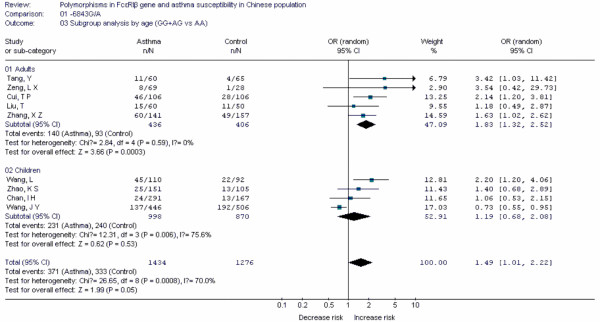
**Forest plot of asthma risk associated with *FcεRIβ *-6843G/A in Chinese population**. Subgroup analysis by age.

**Figure 9:**
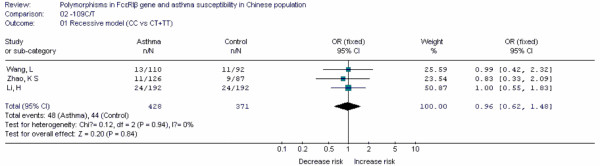
**Forest plot of asthma risk associated with *FcεRIβ *-109C/T in Chinese population**.

**Figure 10:**
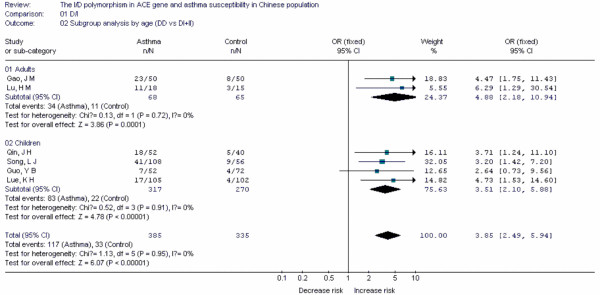
**Forest plot of asthma risk associated with *ACE *D/I in Chinese population**. Subgroup analysis by age.

**Figure 11:**
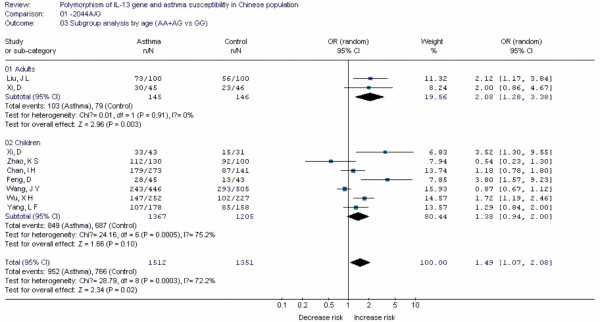
**Forest plot of asthma risk associated with *IL-13 *-2044A/G in Chinese population**. Subgroup analysis by age.

**Figure 12:**
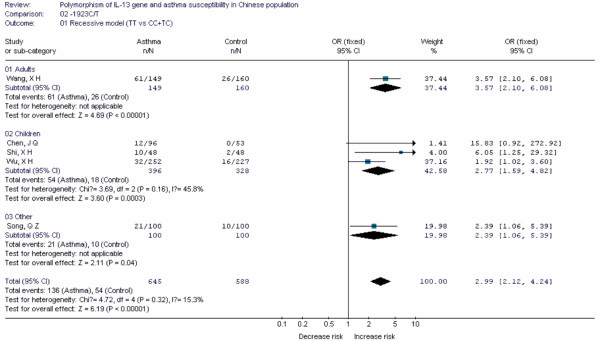
**Forest plot of asthma risk associated with *IL-13 *-1923C/T in Chinese population**.

**Figure 13:**
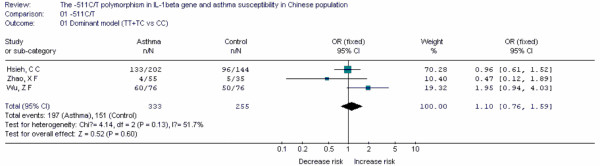
**Forest plot of asthma risk associated with *IL-1β*-511C/T in Chinese population**.

**Figure 14:**
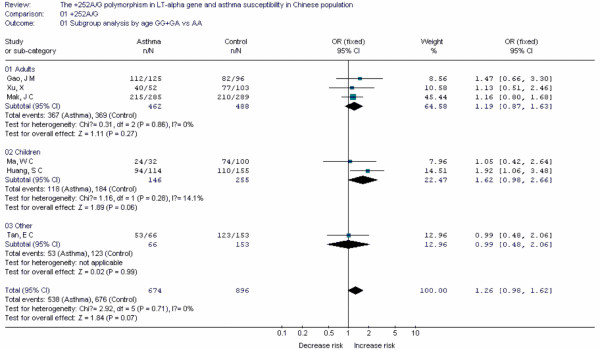
**Forest plot of asthma risk associated with *LT-α *+252A/G in Chinese population**.

**Figure 15:**
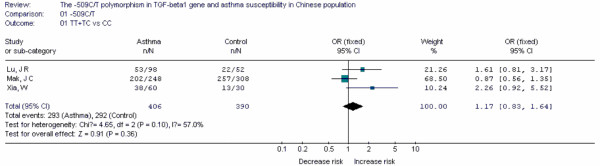
**Forest plot of asthma risk associated with *TGF-β1 *-509C/T in Chinese population**.

**Figure 16:**
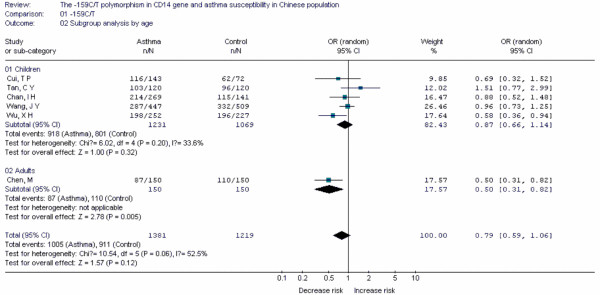
**Forest plot of asthma risk associated with *CD14 *-159C/T in Chinese population**.

**Figure 17:**
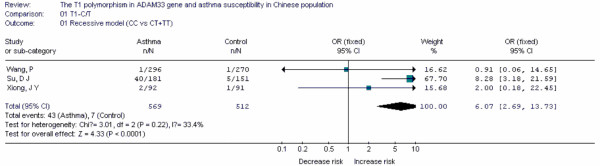
**Forest plot of asthma risk associated with *ADAM33 *T1-C/T in Chinese population**.

**Figure 18:**
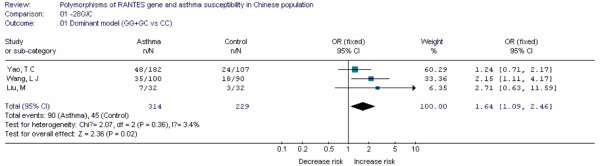
**Forest plot of asthma risk associated with *RANTES *-28G/C in Chinese population**.

**Figure 19:**
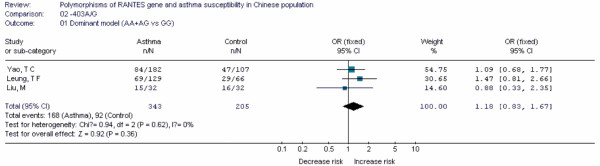
**Forest plot of asthma risk associated with *RANTES *-403A/G in Chinese population**.

Forest plots of each polymorphism can be seen in Figure 2, 3, 4, 5, 6, 7, 8, 9, 10, 11, 12, 13, 14, 15, 16, 17, 18 and 19. In summary, we abstained significant results for seven polymorphisms: *ADAM33 *T1-C/T (OR = 6.07, 95%CI: 2.69-13.73, *Z *= 4.33, *P *< 0.0001), *ACE *D/I(OR = 3.85, 95%CI: 2.49-5.94, *Z *= 6.07, *P *< 0.00001), *FcεRIβ *-6843G/A (OR = 1.49, 95%CI: 1.01-2.22, *Z *= 1.99, *P *= 0.05), *IL-13 *-1923C/T(OR = 2.99, 95%CI: 2.12-4.24, *Z *= 6.19, * P*< 0.00001), *IL-13 *-2044A/G(OR = 1.49, 95%CI: 1.07-2.08, *Z *= 2.34, *P *= 0.02), *RANTES *-28C/G (OR = 1.64, 95%CI: 1.09-2.46, *Z *= 2.36, *P *= 0.02), *TNF-α *-308G/A (OR = 1.42, 95%CI: 1.09-1.85, *Z *= 2.63, *P *= 0.009). These results indicated that these polymorphisms were significant associated with asthma risk in Chinese population. All results for all 18 meta-analyzes are summarized in table 20.

To evaluate the age-specific effects, subgroup analyses were performed by age for polymorphisms which were investigated in a sufficient number of studies(data were available from at least three case-control studies for at least one subgroup). Three subgroups were used: adults, children, others(ages in these case-control studies were not mentioned or mixed with adults and children). Briefly, we obtained significant results from five polymorphisms(*ACE *D/I, *β2-AR *-79G/C, *TNF-α *-308G/A, *IL-4R *-1902G/A and *IL-13 *-1923C/T) in children and five polymorphisms(*ACE *D/I, *FcεRIβ *-6843G/A, *TNF-α *-308G/A, *IL-13 *-1923C/T, *IL-13 *-2044A/G) in adults.

### Publication bias

The Begg's funnel plots and Egger's tests were performed to assess the potential publication bias (Begg's funnel plots can be seen in Additional File 1). The results did not suggest evidence of publication bias except for the *FcεRIβ *-6843G/A polymorphism. Statistical results of Begg's test are summarized in Table 20.

## Discussion

The aim of meta-analysis is to combine results from studies on the same topic and to produce more precise results. The current study is to reveal the roles of genetic variants and their associations with risk of asthma in Chinese population. In summary, we finally identified 18 polymorphisms in 13 genes. Among them, seven polymorphisms (*ADAM33 *T1-C/T, *ACE *D/I, *FcεRIβ *-6843G/A, *IL-13 *-1923C/T, *IL-13 *-2044A/G, *RANTES *-28C/G and *TNF-α *-308G/A) were statistically associated with increased risk of asthma. In order to analysis the age-specific associations, subgroup analysis were performed by age. The *ACE *D/I, *β2-AR *-79G/C, *TNF-α *-308G/A, *IL-4R *-1902G/A and *IL-13 *-1923C/T polymorphisms were found being associated with asthma risk in Chinese children, while the *ACE *D/I, *FcεRIβ *-6843G/A, *TNF-α *-308G/A, *IL-13 *-1923C/T, *IL-13 *-2044A/G polymorphisms were associated with asthma risk in Chinese adults. Given that the data for each polymorphism were from at least three case-control studies, the obtained results could be more precise than results obtained form any individual study.

The *β2-AR *gene is a critical gene in the pathogenesis of asthma. β2-ARs are present on many airway cells, especially in smooth muscle cells which are hyperreactive in asthmatic patients. At present, β2-AR agonists were major methods for treating asthmatic patients. In this meta-analysis, ten case-control studies for *β2-AR *-79G/C and eleven for -46G/A polymorphism were identified. The results indicated the two polymorphisms were not associated with asthma risk in Chinese population. After subgroup analysis by age, the -79G/C polymorphism was associated with decreased risk of asthma in Chinese children. Up to now, three meta-analyses had been performed to investigate the association between polymorphism of *β2-AR *gene and risk of asthma [10-12]. Thakkinstian A[12] found that the heterozygote in -79G/C was associated with decreased risk of asthma in both adults and children. However, we didn't find these associations in Chinese adults, which suggested different roles of this polymorphism may exist in the pathogenesis of asthma in difference age groups. Previous study indicated that the -46G allele enhanced agonist-induced down regulation of the receptor, and the -79G allele might enhance resistance to down regulation. In combination with our results, personalized therapy of asthma patients in different age population with different genetic backgrounds in Chinese population should also be carried out in clinical practices.

The TNF-α gene, encodes a key proinflammatory cytokine in airway, is located on an asthma susceptible region-chromosome 6p. The TNF-α protein plays a central role in inflammation and involves in pathogenesis of asthma. Several polymorphisms have been identified in this gene, such as -308A/G, -238A/G. The -308A/G polymorphism in the promoter may affect the expression of this cytokine, which may affect the occurrence of asthma. In the meta-analysis performed by Gao and colleagues[13], they found the A allele was significant with increased risk of asthma (OR = 1.37, 95%CI = 1.02-1.84 for A vs. G). Consistently, we found the *TNF-α*-308A/G polymorphism was significantly associated with increased risk of asthma (OR = 1.36, 95%CT = 1.13-1.63 for AA+AG vs. GG) in Chinese population. For A vs G, the pooled OR is 1.26 with 95%CI: 1.08-1.47 in this study, which suggested a weaker association between this polymorphism and asthma risk in Chinese population.

*IL-4 *gene is located on chromosome 5q31, it was suggested to be associated with asthma risk, including elevated serum IgE levels and airway hypersensitiveness. A few studies indicated the -589C/T polymorphism in the promoter as a risk factor for asthma, but with inconclusive results. Li and colleagues performed a meta-analysis and found the T allele was associated with decrease risk of asthma(T vs C: OR = 0.86, 95%CI = 0.78-0.94)[14]. However, our results didn't reveal a positive association between this polymorphism and risk of asthma in Chinese. Compared with Li's study, the total number of studies concerning the Chinese population was smaller, which suggested more studies should be carried out to reveal these associations.

IL-4 and IL-13 signal through binding to a receptor complex comprised of the IL-13Rα1 and IL-4Rα with subsequent phosphorylation of JAKs and STAT6[15]. IL-4 receptor plays its role in inflammation through IL-4 and IL-13. The *IL-4 receptor *gene is located on chromosome 16 p12.1-p11.2. Some polymorphisms had been identified as risk factors for asthma, such as -1902G/A and -223G/A. Our results indicated the -1902G/A polymorphism was associated with increased risk of asthma in Chinese children, but not in Chinese adults. The results also indicated the -223G/A polymorphism was not associated with risk of asthma in Chinese population.

The *FcεRIβ *gene is a major candidate gene, involving in the pathogenesis of asthma. It is located on the chromosome 11q13. The -6843G/A polymorphism, leading change in an amino acid sequence at residue 237 from glutamic acid to glycine, is associated with increased IgE levels in atopic asthmatic children. In Chinese population, the -6843G/A polymorphism is the most extensively studied polymorphism in *FcεRIβ *gene. Our study revealed this polymorphism as a risk factor of asthma in Chinese population. Chinese who carry the GG or GA genotype have an 49% increased risk of asthma than AA carriers. Our results also demonstrated the -109C/T polymorphism in this gene was not associated with increased risk of asthma in Chinese population.

Up to date, we first found that *ADAM33 *T1-C/T, *ACE *D/I, *IL-13 *-1923C/T, *RANTES *-28C/G and *IL-13 *-2044A/G polymorphisms were associated with risk of asthma in Chinese population by using meta-analyzes. Some results are similar to other studies performed in other ethnic- groups and some are not. In future, more published results should be included to update and validate these associations in Chinese population.

In this study, the rigorous inclusive criteria made the results more precise. Any study in which genotype distribution of control group divorced from HWE was excluded. In this meta-analysis, 11 polymorphisms were synthesized by using the fixed-effect model, 7 used random-effects model. Because the fixed-effect model is more precise than random effect model, the strength of evidence of *ADAM33 *T1-C/T, *ACE *D/I, *IL-13 *-1923C/T, *RANTES *-28C/G, as risk factors for asthma was greater than that of *FcεRIβ *-6843G/A, *IL-13 *-2044A/G and *TNF-α *-308G/A.

The heterogeneity of clinical information among studies should also be mentioned. Heterogeneity is an important issue when interpreting the results of meta-analysis. Significant heterogeneity existed in overall comparisons in a few meta-analyses, such as *FcεRIβ *-6843G/A. After subgroup analyses by age, the heterogeneity was effectively decreased or removed in adults. Possible explanation may be that differences in etiology may exist in difference age groups. Another important factor contributing to heterogeneity was that homogeneity in either the case and control groups was uncertain. Ideally, all cases and controls in this meta-analysis should be matched for age, sex, atopic status and environmental exposures. However, these issues could not all be explained precisely because of insufficient clinical information for individual person. In addition, because this study is based on population of Chinese descent with the same genetic background, so the similarity of these studies might be very good, despite most studies were conducted in different areas of China.

Some limitations of this meta-analysis should be acknowledged when explaining our results. First, only published articles in the selected electronic databases were included in this study, it may be possible that some studies were not included in those databases or some unpublished studies which had null results, which might bias the results. Second, due to lack of sufficient data, the homogeneity in either the case and control groups was uncertain and data were not stratified by other factors such as atopic status or sex. The tests for gene-environment interactions were not carried out either. Third, publication bias may affect the results. Although *P *values of Begg's test were more than 0.05 in 18 meta-analyses, we could not rule out this possibility, because for some polymorphisms, the included number of studies were relatively small. Third, this study didn't included some polymorphisms with lack of number of studies, or polymorphisms which were not characterized as -A/B for lack of quality analysis for HWE, some polymorphism, such as GSTM1-P/N, or HLA DR1 alleles and MHC alleles were not included, future studies should performed to analysis the effect of these polymorphism in Chinese population.

To our knowledge, this is the first and most comprehensive genetic meta-analysis to date conducted in Chinese descent for any respiratory diseases. In conclusion, this meta-analysis indicated the T1-C/T polymorphism in *ADAM33 *gene, the D/I polymorphism in *ACE *gene, the -6843G/A polymorphism in *FcεRIβ *gene, the -1923C/T polymorphism in *IL-13 *gene, the -2044A/G polymorphism in *IL-13 *gene, the -28C/G polymorphism in *RANTES *gene and the -308G/A polymorphism in *TNF-α *gene are associated with asthma risk in Chinese population. And these results may also implicate in personalized therapy for asthma in Chinese population. In future, more studies should be conducted to investigate the gene-gene and gene-environment interactions between these polymorphisms in Chinese population.

## Abbreviations

ADAM33: A Disintegrin and Metalloprotease 33; FcεRIβ: High-affinity IgE receptor β chain; ACE: Angiotensin-Converting Enzyme; β2-AR: β2-Adrenergic Receptor; IL-4: Interleukin 4; IL-13: Interleukin 13; IL-1β: Interleukin 1β; LT-α: Lymphotoxin-α; RANTES: Regulated upon Activation, Normal T cell Expressed and Secreted; TNF-α: Tumor Necrosis Factor-α; TGF-β1: Transforming Growth Factor β1.

## Competing interests

The authors declare that they have no competing interests.

## Authors' contributions

HF designed the study, provided resources, coordinated the study and directed its implementation; XBL, YGZ and JZ searched the publications, extracted the data and wrote the materials and methods, results; YLX wrote the discussion and checked all data, JH was responsible for data synthesis, CT and CH helped designed the study's analytic strategy, YD edited the manuscript, YYY wrote the introduction. All authors read and approved the final manuscript.

## Supplementary Material

Additional file 1 **Begg's funnel plots for publication bias in selection of studies on asthma susceptibility genes in Chinese**. Figure S1 Begg's funnel plots for publication bias in selection of studies on *β2-AR *-46G/A polymorphism. Figure S2 Begg's funnel plots for publication bias in selection of studies on *β2-AR *-79G/C polymorphism. Figure S3 Begg's funnel plots for publication bias in selection of studies on *IL-4R *-1902G/A polymorphism. Figure S4 Begg's funnel plots for publication bias in selection of studies on *IL-4R *-223G/A polymorphism. Figure S5 Begg's funnel plots for publication bias in selection of studies on *IL-4 *-589C/T polymorphism. Figure S6 Begg's funnel plots for publication bias in selection of studies on *TNF-α *-308A/G polymorphism. Figure S7 Begg's funnel plots for publication bias in selection of studies on *FcεRIβ *-6843G/A polymorphism. Figure S8 Begg's funnel plots for publication bias in selection of studies on *FcεRIβ *-109C/T polymorphism. Figure S9 Begg's funnel plots for publication bias in selection of studies on *ACE *D/I polymorphism. Figure S10 Begg's funnel plots for publication bias in selection of studies on *IL-13 *-2044A/G polymorphism. Figure S11 Begg's funnel plots for publication bias in selection of studies on *IL-13 *-1923C/T polymorphism. Figure S12 Begg's funnel plots for publication bias in selection of studies on *IL-1β*-511C/T polymorphism. Figure S13 Begg's funnel plots for publication bias in selection of studies on *LT-α *+252A/G polymorphism. Figure S14 Begg's funnel plots for publication bias in selection of studies on *TGF-β1 *-509C/T polymorphism. Figure S15 Begg's funnel plots for publication bias in selection of studies on *CD14 *-159C/T polymorphism. Figure S16 Begg's funnel plots for publication bias in selection of studies on *ADAM33 *T1-C/T polymorphism. Figure S17 Begg's funnel plots for publication bias in selection of studies on *RANTES *-28G/C polymorphism. Figure S18 Begg's funnel plots for publication bias in selection of studies on *RANTES *-403A/G polymorphism Click here for file
