# Supplementary material for: Asthma susceptible genes in Chinese population: A meta-analysis
Source: Respir Res. 2010 Sep 24;11(1):129. doi: 10.1186/1465-9921-11-129 (PMC2955661; doi:10.1186/1465-9921-11-129)

Additional file 1

Title: Begg’s funnel plots for publication bias in selection of studies on asthma susceptibility genes in Chinese

Figure S1 Begg’s funnel plots for publication bias in selection of studies on *β2-AR* -46G/A polymorphism


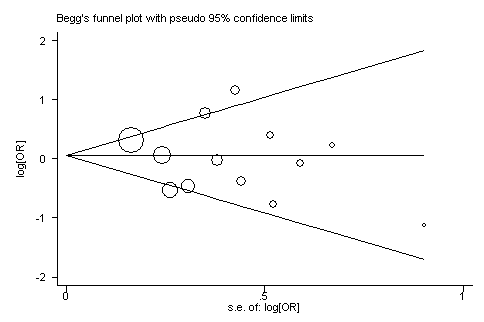


Figure S2 Begg’s funnel plots for publication bias in selection of studies on *β2-AR* -79G/C polymorphism


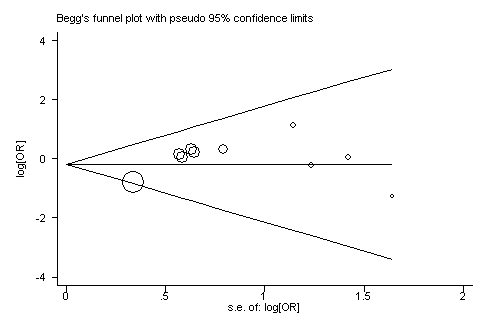


Figure S3 Begg’s funnel plots for publication bias in selection of studies on *IL-4R* -1902G/A polymorphism


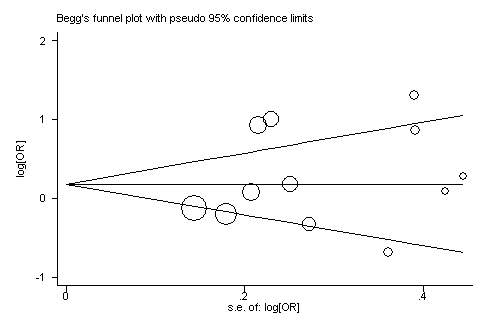


Figure S4 Begg’s funnel plots for publication bias in selection of studies on *IL-4R* -223G/A polymorphism


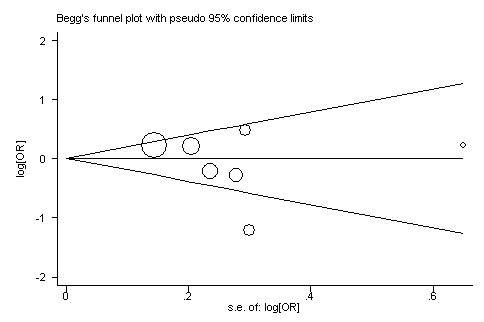


Figure S5 Begg’s funnel plots for publication bias in selection of studies on *IL-4* -589C/T polymorphism


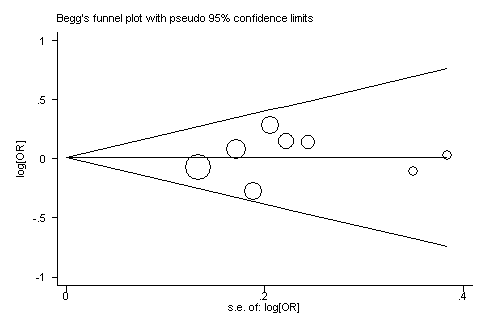


Figure S6 Begg’s funnel plots for publication bias in selection of studies on  *TNF-α* -308A/G polymorphism


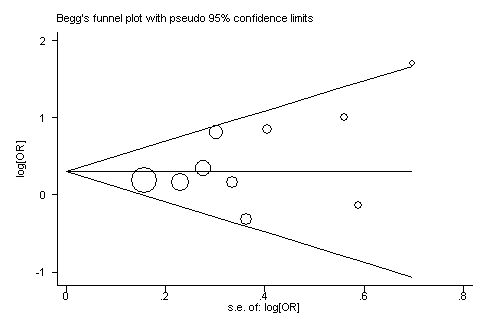


Figure S7 Begg’s funnel plots for publication bias in selection of studies on *FcεRIβ* -6843G/A polymorphism


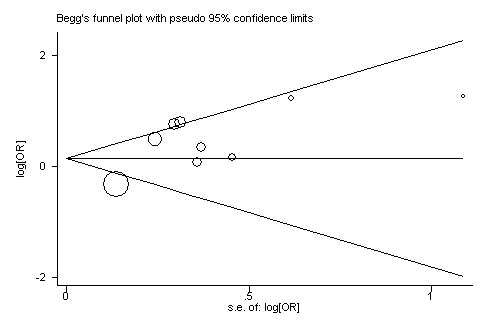


Figure S8 Begg’s funnel plots for publication bias in selection of studies on *FcεRIβ* -109C/T polymorphism


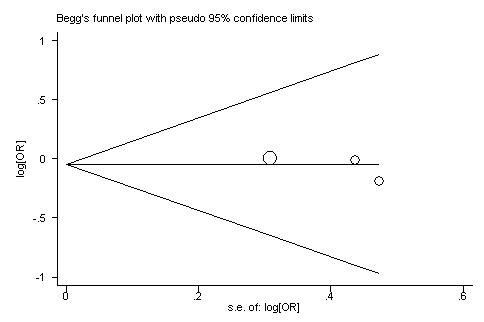


Figure S9 Begg’s funnel plots for publication bias in selection of studies on *ACE* D/I polymorphism


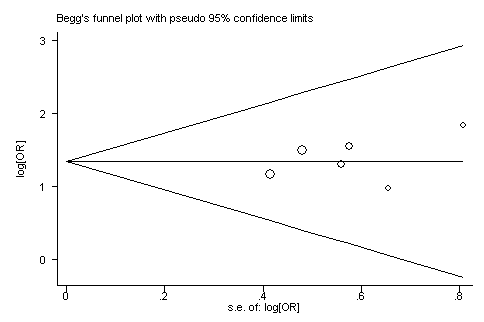


Figure S10 Begg’s funnel plots for publication bias in selection of studies on *IL-13* -2044A/G polymorphism


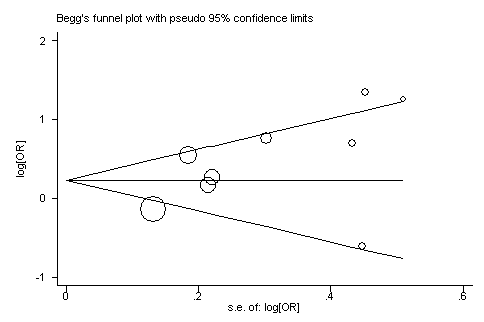


Figure S11 Begg’s funnel plots for publication bias in selection of studies on *IL-13* -1923C/T polymorphism


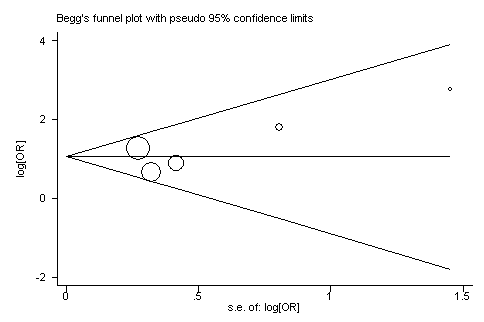


Figure S12 Begg’s funnel plots for publication bias in selection of studies on *IL-1β*-511C/T polymorphism


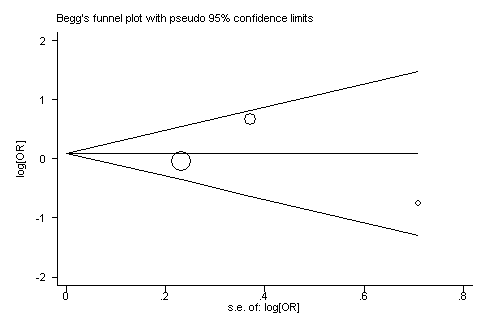


Figure S13 Begg’s funnel plots for publication bias in selection of studies on *LT-α* +252A/G polymorphism


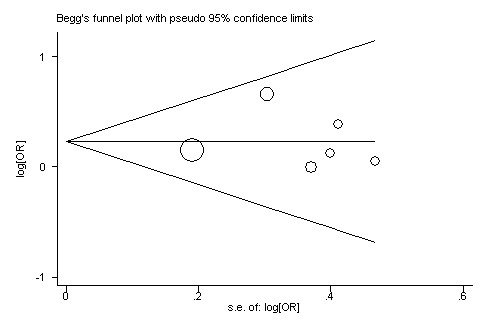


Figure S14 Begg’s funnel plots for publication bias in selection of studies on *TGF-β1* -509C/T polymorphism


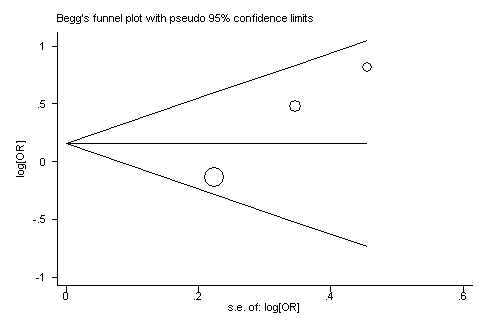


Figure S15 Begg’s funnel plots for publication bias in selection of studies on *CD14* -159C/T polymorphism


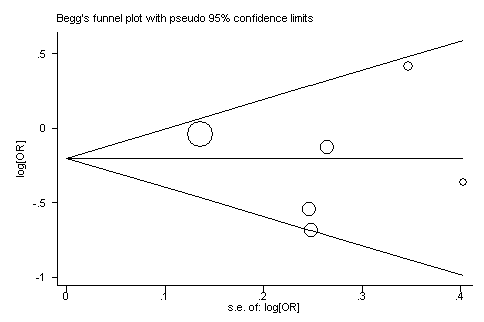


Figure S16 Begg’s funnel plots for publication bias in selection of studies on *ADAM33* T1-C/T polymorphism


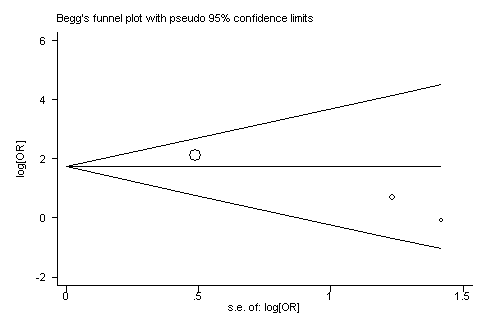


Figure S17 Begg’s funnel plots for publication bias in selection of studies on RANTES -28G/C polymorphism


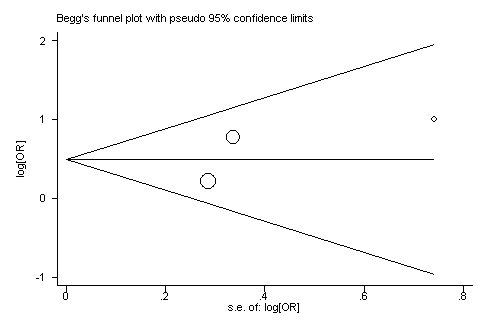


Figure S18 Begg’s funnel plots for publication bias in selection of studies on RANTES -403A/G polymorphism


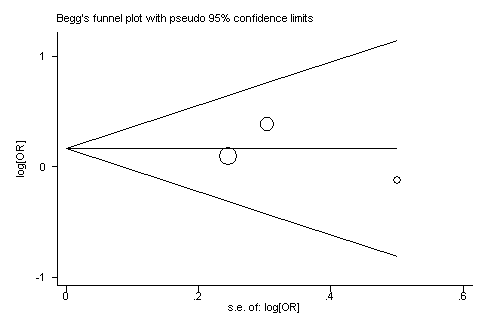

Supplement: Additional file 1 — Begg's funnel plots for publication bias in selection of studies on asthma susceptibility genes in Chinese. Figure S1 Begg's funnel plots for publication bias in selection of studies on β2-AR -46G/A polymorphism. Figure S2 Begg's funnel plots for publication bias in selection of studies on β2-AR -79G/C polymorphism. Figure S3 Begg's funnel plots for publication bias in selection of studies on IL-4R -1902G/A polymorphism. Figure S4 Begg's funnel plots for publication bias in selection of studies on IL-4R -223G/A polymorphism. Figure S5 Begg's funnel plots for publication bias in selection of studies on IL-4 -589C/T polymorphism. Figure S6 Begg's funnel plots for publication bias in selection of studies on TNF-α -308A/G polymorphism. Figure S7 Begg's funnel plots for publication bias in selection of studies on FcεRIβ -6843G/A polymorphism. Figure S8 Begg's funnel plots for publication bias in selection of studies on FcεRIβ -109C/T polymorphism. Figure S9 Begg's funnel plots for publication bias in selection of studies on ACE D/I polymorphism. Figure S10 Begg's funnel plots for publication bias in selection of studies on IL-13 -2044A/G polymorphism. Figure S11 Begg's funnel plots for publication bias in selection of studies on IL-13 -1923C/T polymorphism. Figure S12 Begg's funnel plots for publication bias in selection of studies on IL-1β-511C/T polymorphism. Figure S13 Begg's funnel plots for publication bias in selection of studies on LT-α +252A/G polymorphism. Figure S14 Begg's funnel plots for publication bias in selection of studies on TGF-β1 -509C/T polymorphism. Figure S15 Begg's funnel plots for publication bias in selection of studies on CD14 -159C/T polymorphism. Figure S16 Begg's funnel plots for publication bias in selection of studies on ADAM33 T1-C/T polymorphism. Figure S17 Begg's funnel plots for publication bias in selection of studies on RANTES -28G/C polymorphism. Figure S18 Begg's funnel plots for publication bias in selection of studies on RANTES - [file 1465-9921-11-129-S1.DOC]
